# Cerebral autoregulation and response to intravenous thrombolysis for acute ischemic stroke

**DOI:** 10.1038/s41598-020-67404-9

**Published:** 2020-06-29

**Authors:** Ricardo C. Nogueira, Man Y. Lam, Osian Llwyd, Angela S. M. Salinet, Edson Bor-Seng-Shu, Ronney B. Panerai, Thompson G. Robinson

**Affiliations:** 1 Neurology Department, School of Medicine, Hospital das Clinicas, University of São Paulo, São Paulo, 01246-904 Brazil; 2 Department of Cardiovascular Sciences, Cerebral Haemodynamics in Ageing and Stroke Medicine Research Group, University of Leicester, Leicester, LE2 7LX UK; 3 NIHR Leicester Biomedical Research Centre, University of Leicester, Leicester, LE3 9QP UK; 4 Department of Neurology, Hospital Nove de Julho, São Paulo, Brazil

**Keywords:** Stroke, Biomedical engineering

## Abstract

We hypothesized that knowledge of cerebral autoregulation (CA) status during recanalization therapies could guide further studies aimed at neuroprotection targeting penumbral tissue, especially in patients that do not respond to therapy. Thus, we assessed CA status of patients with acute ischemic stroke (AIS) during intravenous r-tPA therapy and associated CA with response to therapy. AIS patients eligible for intravenous r-tPA therapy were recruited. Cerebral blood flow velocities (transcranial Doppler) from middle cerebral artery and blood pressure (Finometer) were recorded to calculate the autoregulation index (ARI, as surrogate for CA). National Institute of Health Stroke Score was assessed and used to define responders to therapy (improvement of ≥ 4 points on NIHSS measured 24–48 h after therapy). CA was considered impaired if ARI < 4. In 38 patients studied, compared to responders, non-responders had significantly lower ARI values (affected hemisphere: 5.0 vs. 3.6; unaffected hemisphere: 5.4 vs. 4.4, *p* = 0.03) and more likely to have impaired CA (32% vs. 62%, *p* = 0.02) during thrombolysis. In conclusion, CA during thrombolysis was impaired in patients who did not respond to therapy. This variable should be investigated as a predictor of the response to therapy and to subsequent neurological outcome.

## Introduction

The key objective of current acute ischemic stroke (AIS) treatment is based on rapid blood flow restoration by thrombolysis, using intravenous recombinant tissue plasminogen activator (r-tPA), and/or mechanical arterial recanalization techniques^1-3^. Several factors predict stroke outcome including age, initial stroke severity, arterial blood pressure (BP), site of occlusion, collaterals, and others^4,5^. Nevertheless, complete or partial successful recanalization may not necessarily result in favorable outcome, with a number of predictors hypothesized^6,7^. In particular, BP control may affect penumbral lesion size, with an optimal strategy still lacking evidence^8–10^. Therapeutic BP manipulation may further impact on microvascular autoregulatory failure, as a consequence of an increase in lactate and free oxygen radicals in the occluded and/or reperfused tissues^11^.

Cerebral autoregulation (CA) refers to a set of physiological mechanisms that maintain the constancy of cerebral blood flow (CBF) despite wide variations in arterial BP. CA can be impaired within the first hours of ischemic stroke onset^11^; as a consequence, BP control may be important for improving both the ischemic area in the brain and clinical outcome. Therefore, assessment of CA during recanalization therapy for AIS is relevant, and may influence future strategies for personalized BP control and associated neuroprotection.

The aims of the present study were to assess CA status of responder and non-responder AIS patients to intravenous r-tPA during the therapy and after 24–48 h, and to test the hypothesis that CA during thrombolysis is associated with early response to therapy.

## Results

Forty-five patients (34 from São Paulo, 11 from Leicester) met the inclusion criteria. Seven patients were excluded due to absence of a temporal window or poor quality of acquired data from both hemispheres, leaving 38 patients for further analysis. From these, eight hemisphere’s data (5 affected and 3 unaffected) during thrombolysis and 3 (2 affected and 1 unaffected) after 24–48 h had to be discarded due to proximal occlusion of MCA (3 patients) or poor quality of data (5 patients). In addition, in one affected hemisphere, data were not included at both time-points because the patient had a vertebrobasilar stroke and the data from both hemispheres were averaged. Demographics and patient characteristics for responders and non-responders groups are given in Table 1, with a higher 24–48 h NIHSS in the non-responder group being the only significant difference.

**Table 1:** Demographic and systemic hemodynamic data.

	Responders (n = 24)	Non-responders (n = 14)
Age, years	68.64 (11.95)	65.04 (13.23)
NIHSS initial	12 (4–20)	11 (4–24)
NIHSS end	10 (0–20)	8 (3–20)
NIHSS 24–48	4 (0–14)*	12 (3–30)
Symptomatic hemorrhage (%)	0	1 (7%)
Stroke onset to thrombolysis time, min	175 (52)	195 (51)
EtCO_2_, mmHg	37.57 (10.85)	36.26 (6.43)
Mean BP, mmHg	90.18 (11.61)	96.11 (15.33)
Systolic BP, mmHg	131.09 (17.53)	131.03 (19.54)
Diastolic BP, mmHg	68.94 (12.79)	75.35 (14.68)
Carotid occlusion (%)	3 (12%)	4 (28%)
Diabetes (%)	5 (21%)	6 (42%)
Atrial fibrillation (%)	2 (8%)	2 (14%)

Data are mean (standard deviation) or median (range).

*EtCO*_*2*_ End tidal CO_2_, *BP *blood pressure, *NIHSS *National Institutes of Health Stroke Scale.

*p < 0.05 versus non-responders.

**Figure 1:**
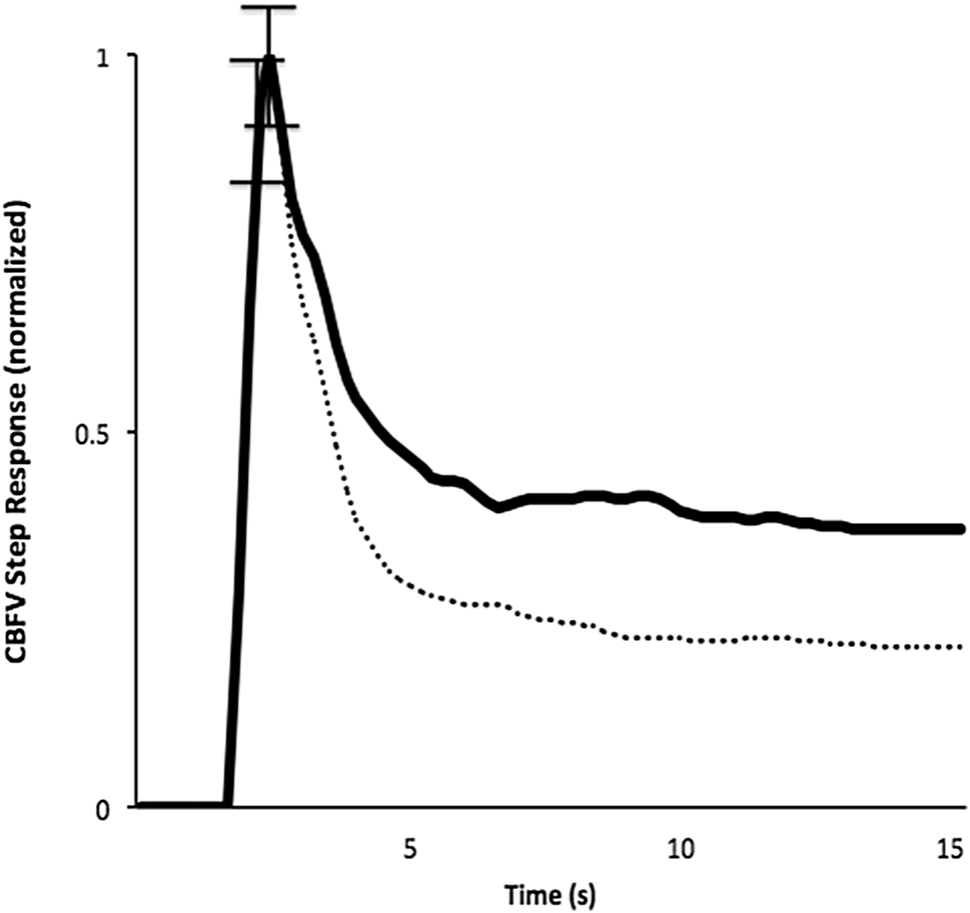
Population average CBFV step response from responders and non-responders during thrombolysis. Curves are average from both hemispheres. Error bars correspond to the largest ± 1 SEM at the point of occurrence.

Regarding the cerebral hemodynamic data, CBFV from both groups was significantly higher at 24–48 h than during thrombolysis (Table 2). ARI during thrombolysis was significantly lower in non-responders in both AH and UH, though no significant differences were seen at 24–48 h (Table 2, Fig. 1). No significant differences were seen in values for phase or gain between responders and non-responders during thrombolysis or at 24–48 h in either the VLF or LF bands (Table 2).

**Table 2:** Cerebral hemodynamic patient data, dichotomized by responders and non-responders.

	Responders				Non-responders			
	Thrombolysis		24–48 h		Thrombolysis		24–48 h	
	Affected side	Unaffected side	Affected side	Unaffected side	Affected side	Unaffected side	Affected side	Unaffected side
CBFV (cm/s)	41.35 (4.60)†	47.42 (3.53)†	47.75 (4.43)	48.29 (3.10)	39.73 (4.78)†	48.79 (5.00)†	46.82 (4.21)	49.77 (4.39)
ARI	5.01 (0.47)*	5.42 (0.44)*	4.44 (0.37)	4.89 (0.37)	3.68 (0.58)	4.41 (0.58)	4.40 (0.51)	5.25 (0.49)
Gain (cm s^−1^ mm Hg^−1^), VLf	0.548 (0.071)	0.514 (0.067)	0.593 (0.069)	0.563 (0.069)	0.432 (0.088)	0.557 (0.087)	0.532 (0.094)	0.605 (0.091)
Gain (cm s^−1^ mm Hg^−1^), Lf	0.732 (0.086)	0.638 (0.081)	0.740 (0.066)	0.663 (0.066)	0.452 (0.106)	0.668 (0.105)	0.607 (0.090)	0.655 (0.087)
Phase (radians), VLf	0.772 (0.097)	0.830 (0.091)	0.629 (0.101)	0.813 (0.101)	0.475 (0.118)	0.502 (0.118)	0.600 (0.140)	0.754 (0.134)
Phase (radians), Lf	0.644 (0.066)	0.709 (0.061)	0.539 (0.086)	0.562 (0.080)	0.528 (0.085)	0.601 (0.085)	0.543 (0.110)	0.572 (0.110)

Data are mean (SE).

*CBFV* cerebral blood flow velocity, *ARI* autoregulation index, *VLF* very low frequency, *LF* low frequency.

^†^*p* < 0.05 versus 24–48 h for responders and non-responders; *p < 0.05 versus non-responders during thrombolysis.

**Figure 2:**
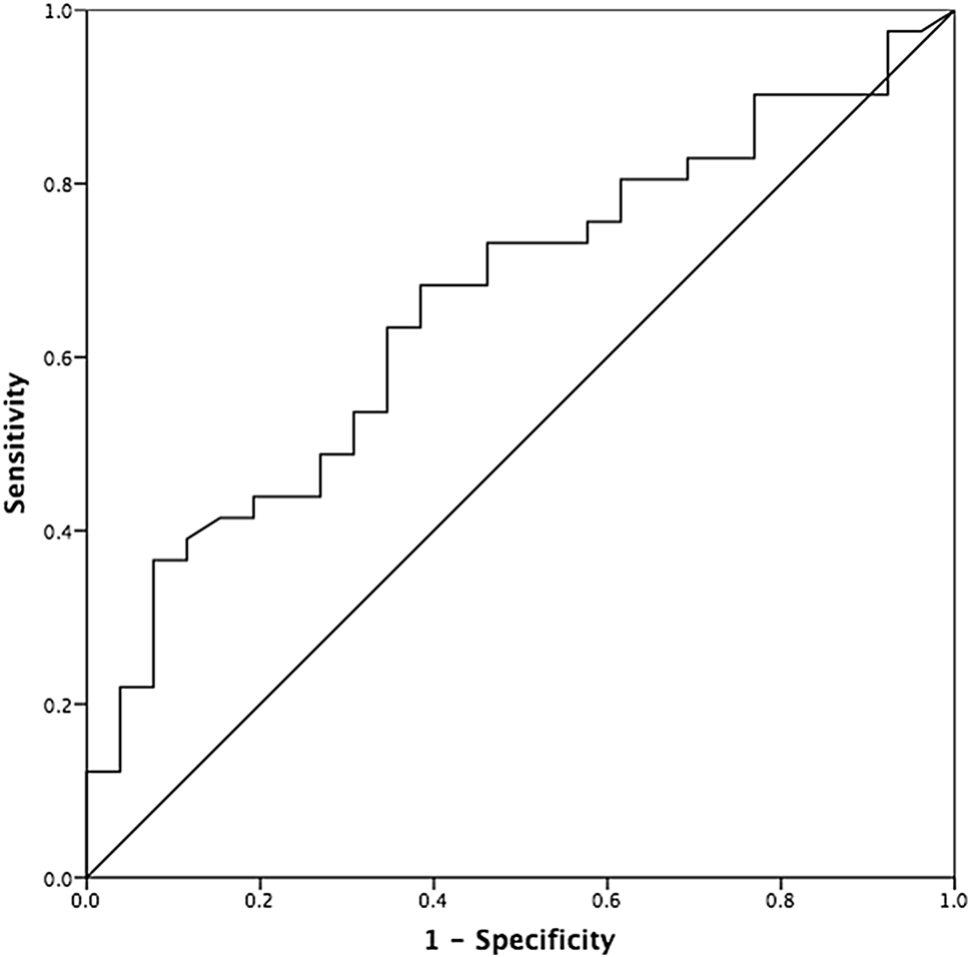
Receiver operator characteristic curve of ARI as predictor of response to therapy.

The number of AIS patients with impaired ARI during thrombolysis, defined by ARI < 4, was significantly greater in the non-responder group (Table 3), but again no significant differences were seen at 24–48 h. ROC analysis confirmed that ARI during thrombolysis predicts good response to therapy (AUC 0.66, *p* = 0.02; Fig. 2), and an ARI cut-off value of 4.0 had the best sensitivity and specificity (0.68 and 0.62, respectively).

**Table 3:** Dichotomized ARI for responders and non-responders during thrombolysis and 24–48 h after.

	Thrombolysis				*p* value*
	Responders		Non-responders		
	Affected	Unaffected	Affected	Unaffected	
ARI < 4	7 (17%)	6 (15%)	8 (31%)	8 (31%)	0.02
ARI ≥ 4	12 (29%)	16 (39%)	5 (19%)	5 (19%)	

	24–48 h				*p* value
	Responders		Non-responders		
	Affected	Unaffected	Affected	Unaffected	
ARI < 4	9 (20%)	6 (13%)	7 (27%)	6 (23%)	0.20
ARI ≥ 4	14 (30%)	17 (37%)	5 (19%)	8 (31%)	

Data are n (% of total for responders and non-responders).

*p value for Fisher exact test of sum of hemispheres (affected and unaffected) for both groups (responders vs. non-responders).

## Discussion

To our knowledge, this is the first study reporting the status of CA during intravenous thrombolytic therapy. The main finding is that ARI was lower during thrombolytic therapy in non-responders, and thus may be considered as a potential evaluation tool to predict response to therapy and also could be important in planning neuroprotective strategies for the ischemic penumbra.

Due to its limited energy reserve, the brain is highly dependent on stable blood flow, and has evolved a number of protective mechanisms to maintain cerebral perfusion^12^. However, these regulatory mechanisms may be impaired during and following ischemia^12^, and this has particular importance for BP management in the hyperacute period following AIS, when associated BP disturbances may increase the risk of further damage^13-15^. Few studies have assessed CA acutely (< 24 h) following AIS^11,13,16-21^, and only one study in a small sample of AIS patients with major anterior circulation stroke (NIHSS ≥ 10) undergoing thrombolytic therapy, was performed an average of 20 h post-symptom onset. This study reported that severe CA impairment in the AH (assessed by Mx index and phase shift) was associated with poor outcome^11^. Though our study found less severe impairment, it has extended these observations to a broader AIS population (NIHSS 4–24), for both AH and UH and with recordings undertaken during thrombolysis providing a potential opportunity for future intervention at an earlier stage in the evolution of the ischemic penumbra. In addition, previous studies have demonstrated that CA could be associated with infarction size^13,16,17^ but it is not clear if the ischemic lesion determines the CA impairment or vice versa; although our study did not evaluate infarct volume, the difference of CA in the very early phase amongst the two groups, (responders x non-responders) with no difference after 24–48 h, suggests that in our population, CA impairment was not influenced by infarct extension. We also could not demonstrate a direct link between intracranial haemorrhage and CA during thrombolytic therapy because this complication only occurred in one patient; however it is plausible to assume that impaired CA during therapy could have impacted on this complication as demonstrated in previous work^18^.

Whilst the main goal of acute stroke treatment is recanalization of the occluded vessel, a significant percentage of patients do not have good neurological outcome despite successful recanalization, so-called ‘futile recanalization’^6,7^. Different hypotheses have been put forward trying to explain this phenomenon, such as collateral status and/or microvascular occlusion^7^; however it also seems plausible to consider the role of impairment of CBF regulatory mechanisms in worsening neurological outcome in patients with futile recanalization. For example, CA impairment could lead to further penumbral damage secondary to reperfusion, as reported in a previous study within 6 h of AIS^18^. Interestingly, in the pivotal NINDS trial^1^, there was no difference in early neurologic improvement amongst treated and placebo patient groups; this finding corroborates our hypothesis that there are other factors apart from recanalization that influences clinical outcome in the early stages after ischemic stroke. Thus, our findings are important to drive the investigation of strategies aimed to preserve the penumbral tissue and improve neurological recovery, irrespective of successful recanalization or not.

The ROC analysis in our study revealed that a cut-off value of 4.0 had the best sensitivity and specificity for predicting response to therapy and is in agreement with previous publications suggesting an ARI < 4 to define impaired CA^22,23^. This cut-off value is a new finding in a stroke population, and should be further investigated and replicated in larger studies to be implemented as a valid assessment tool for detection of CA impairment.

Our study has a number of limitations. First, there is a known limitation of TCD when used as a surrogate of CBF^24^. Secondly, we included patients with carotid stenosis, which may also impair CA, though we found no difference in the prevalence or severity of carotid stenosis between responders and non-responders. Thirdly, there was no evaluation of CA before thrombolysis, therefore it was not possible to assess if pre-existing CA impairment can predict poor response despite recanalization; however, it would be challenging, and potentially unethical, to delay treatment to allow pre-thrombolysis CA assessment. Fourthly, CA assessment was not possible during thrombolysis in patients with proximal MCA occlusion (n = 3) and the effects of recanalization, intracranial stenosis and stroke mechanism were not accounted for; this may have over- or under-estimated the predictive effect of impaired peri-thrombolysis ARI. In addition, this study only included thrombolysis-treated patients and not those with mechanical recanalization therapies. Nonetheless, this therapy remains in limited use in both developed and developing countries^25,26^ and it may be possible that this group of patients with higher rates of recanalization have greater influence of CA status on neurologic outcome. Noteworthy, the use of medical reperfusion is increasing in stroke patients with no major vessel occlusion, after publication of trials in wake-up stroke and extended therapeutic window^27,28^. Fifthly, our study investigated only the early response to therapy; thus, the effects of CA in long-term neurologic outcome should be the object of future studies. Finally, the number of patients included in this study is relatively small with heterogeneity of patients and the results obtained are hypothesis generating; in addition it could be argued that the inclusion of one patient with vertebrobasilar stroke (responder group) may have biased the results. We intentionally included this subgroup to try to demonstrate that CA change is more a global than a local phenomenon. Removing this patient from the analysis did not change our results.

In conclusion, the present study has revealed that impaired CA in the very early phase of AIS increases the likelihood of poor response to thrombolytic recanalization therapy, as assessed by NIHSS score at 24–48 h. Based on our results, further studies with larger populations, including different stroke mechanisms and severities, should be planned to corroborate these findings. If our findings are replicated in larger studies, strategies to preserve CBF after recanalization therapies should be implemented to minimise secondary damage in patients with CA impairment.

## Methods

This was a collaborative research project between Hospital das Clinicas, São Paulo University Medical School, Sao Paulo, Brazil, and the Cerebral Haemodynamics in Ageing and Stroke Medicine (CHiASM) Group at the University of Leicester, Leicester, United Kingdom. The researchers involved in the data collection were trained at the same laboratory (Department of Cardiovascular Sciences, University of Leicester, UK) and used a standard protocol for data collection and analysis. The local ethics committee of the University of Sao Paulo and University of Leicester approved the study and informed consent was obtained in compliance with local ethics committee regulations. Both study centres applied the same inclusion criteria: AIS eligible for r-tPA therapy, aged ≥ 18 years, no premorbid disability, ability to monitor systemic and cerebral hemodynamic data without interfering with thrombolytic therapy and any related procedures, and informed consent (or relative assent). Exclusion criteria were: ineligibility for r-tPA thrombolysis and absence of an acoustic window for transcranial Doppler ultrasound (TCD) monitoring.

National Institutes of Health Stroke Scale (NIHSS) scores were measured by neurologists blinded to cerebral hemodynamic data at the following time-points: (1) before therapy (NIHSS_initial_), (2) immediately at the end of therapy (NIHSS_end_), and (3) after 24–48 h (NIHSS_24–48_). The scale was used to assess the early response to therapy, which was defined as improvement of ≥ 4 points on NIHSS_24–48_^1^.

The same protocol for monitoring systemic and cerebral hemodynamic data was used for all studies. Briefly, beat-to-beat BP was recorded continuously using a Finapres or Finometer device (FMS, Finapres Measurement Systems, Arnhem, Netherlands). Heart rate (HR) was recorded using a 3-lead electrocardiogram (ECG) and end-tidal CO_2_ (etCO_2_) was measured via nasal prongs (Salter Labs) by an infrared capnograph (Capnocheck Plus and Transmai MX-200 in Leicester and São Paulo, respectively). Bilateral insonation of the middle cerebral arteries (MCA) was performed using TCD (Viasys Companion III, Viasys Healthcare, and Doppler box, DWL, respectively, for Leicester and São Paulo) with 2 MHz probes, which were secured in place using a head-frame. Hemispheres were classified as affected (AH, side with ischemia) and unaffected (UH, side without ischemia), based on clinical symptoms and confirmed retrospectively with control imaging. If ischemia related to the vertebrobasilar system, both sides were considered as unaffected and they were averaged. Data were collected: (1) during thrombolysis, within the last 30 min of r-tPA infusion; and (2) 24–48 h after the treatment.

### Assessment of dynamic cerebral autoregulation

Data were simultaneously recorded onto a data acquisition system (PHYSIDAS, Department of Medical Physics, University Hospitals of Leicester) for subsequent off-line analysis. Mean BP and CBF velocity (CBFV) values were calculated for each cardiac cycle. Beat-to-beat data were spline interpolated and resampled at 5 samples/s to produce signals with a uniform time-base.

Dynamic CA was calculated by transfer function analysis using spontaneous fluctuations of mean BP as input and corresponding changes in CBFV as output as described previously^24^; then the frequency-dependent estimates of phase were averaged for the very low- (VLF, 0.02–0.07 Hz) and low-frequency (LF, 0.07–0.20 Hz) ranges according to previous guidelines^29^. The autoregulation index (ARI) was extracted by using the best least-squares fit between the CBFV step response, and one of the 10 model ARI curves proposed by Tiecks et al.^30^. To evaluate the frequency of impaired CA amongst the two groups ARI values were dichotomized into impaired (ARI < 4) and unimpaired CA (ARI ≥ 4)^22^.

### Statistical analysis

Statistical software SPSS version 20 was used for all statistical tests. Mean values of each variable were calculated from the entire baseline recording. Tests of normality were performed using the Shapiro–Wilk normality test. To test for differences between AH & UH from responders and non-responders during thrombolysis, and 24–48 h after, a general linear model was used with the following factors: time (thrombolysis × 24–48 h), side (AH x UH), and response (responders × non-responders). Post-hoc comparisons were performed when appropriate, and Bonferroni correction was applied to multiple comparisons. Receiver operator characteristic curve (ROC) analysis was performed to test the prognostic value of ARI in this cohort. Differences amongst categorical data were assessed with Fisher’s exact test and a *p* value < 0.05 indicating statistical significance.

## Data Availability

The datasets generated during and/or analysed during the current study are available from the corresponding author on reasonable request.
